# USP30 inhibition augments mitophagy to prevent T cell exhaustion

**DOI:** 10.1126/sciadv.adv6902

**Published:** 2025-08-15

**Authors:** Ruohan Zhang, Fengxia Gao, Jianying Li, Jiacheng Jin, Kangxuan Chen, Samhita Chaudhuri, Zhiwei Liao, Tong Xiao, Yang Xu, Haitao Wen, Kai He, Zihai Li, Gang Xin, Nuo Sun

**Affiliations:** ^1^ Department of Physiology and Cell Biology, The Ohio State University College of Medicine, Columbus, OH, USA.; ^2^ Pelotonia Institute for Immuno-oncology, The James Comprehensive Cancer Center, The Ohio State University, Columbus, OH, USA.; ^3^ Department of Microbial Infection and Immunity, The Ohio State University College of Medicine, Columbus, OH, USA.; ^4^ Dorothy M. Davis Heart and Lung Research Institute, The Ohio State University Wexner Medical Center, Columbus, OH, USA.

## Abstract

The exhaustion of tumor-infiltrating CD8^+^ T cells poses a substantial challenge in cancer immunotherapy, with mitochondrial health essential for sustaining T cell functionality. Mitophagy, a critical process for mitochondrial quality control, is severely impaired in exhausted CD8^+^ T cells, yet the underlying mechanisms remain unclear. We identified ubiquitin-specific protease 30 (USP30), a mitochondrial deubiquitinase that inhibits mitophagy, as a key factor up-regulated in exhausted CD8^+^ T cells. Notably, prolonged antigen stimulation triggers the T cell receptor and nuclear factor of activated T cell 1 signaling, which drives the transcriptional up-regulation of USP30. Excitingly, our interventions targeting USP30 through genetic deletion or pharmacological inhibition effectively restored mitophagy, improved mitochondrial fitness, and rejuvenated CD8^+^ T cell effector functions. These interventions reinvigorated antitumor responses and markedly suppressed tumor growth. Our findings establish USP30 as a critical regulator of mitophagy and a promising therapeutic target for reversing T cell exhaustion and enhancing the efficacy of cancer immunotherapy.

## Teaser

USP30 inhibition restores mitophagy and reverses CD8^+^ T cell exhaustion to boost antitumor immunity.

## INTRODUCTION

CD8^+^ T cells play a critical role in immune surveillance and cytotoxic response against cancer cells (*1*). However, one of the major challenges in cancer immunotherapy is the functional decline of tumor-infiltrating CD8^+^ T cells (*2*). Hallmark features of this exhaustion process, such as impaired self-renewal, increased expression of inhibitory receptors, and diminished cytotoxic function, are driven by the suppressive mechanisms within the tumor microenvironment (TME) (*3*–*5*). Recent studies have highlighted mitochondrial dysfunction as a key driver of T cell exhaustion (*6*–*8*). Exhausted T cells exhibit notable mitochondrial abnormalities, including increased mitochondrial fragmentation, loss of mitochondrial membrane potential (MMP; depolarization), and the accumulation of dysfunctional mitochondria (*6*, *9*). These alterations are coupled with elevated reactive oxygen species production and impaired oxidative phosphorylation, leading to a decline in adenosine 5′-triphosphate production. This mitochondrial deficit limits T cell proliferation, impairs effector function, and exacerbates exhaustion during chronic antigenic stimulation (*6*, *10*). Despite these observations, the precise mechanisms underlying the loss of mitochondrial integrity and quality in exhausted T cells remain poorly understood. Both animal studies and human clinical trials have demonstrated that tumor-infiltrating CD8^+^ T cells with healthier mitochondria are less exhausted and exhibit improved functionality, which is associated with better treatment outcomes (*11*). Therefore, strategies aimed at improving mitochondrial function in CD8^+^ T cells have emerged as promising therapeutic approaches, with recent studies showing encouraging results (*10*, *11*).

Mitophagy, the selective removal of damaged or dysfunctional mitochondria through autophagy, is a critical mitochondrial quality control mechanism (*12*, *13*). Dysregulation of mitophagy has been increasingly linked to a broad spectrum of pathological conditions and aging (*14*, *15*). In neurodegenerative diseases such as Parkinson’s and Alzheimer’s, impaired mitophagy leads to the accumulation of defective mitochondria, promoting oxidative stress and neuronal degeneration (*14*, *16*). Similarly, in metabolic and cardiovascular disorders, defective mitophagy contributes to bioenergetic failure and increased cellular stress (*17*). Aging is also associated with a progressive decline in mitophagic activity, which exacerbates mitochondrial dysfunction, chronic inflammation, and stem cell exhaustion (*14*, *15*, *18*). Although mitophagy is recognized as a critical process in various diseases and aging, its specific role in T cells has only recently begun to be elucidated. Growing evidence indicates that mitophagy is essential for maintaining mitochondrial integrity, promoting efficient energy production, and sustaining overall metabolic balance in T cells (*19*, *20*). Under conditions of mitochondrial stress, such as nutrient deprivation in the TME, mitophagy plays a crucial role in preventing the accumulation of damaged mitochondria that could, otherwise, compromise cellular function (*21*). Recent studies suggest that impaired mitophagy may lead to the accumulation of dysfunctional, depolarized mitochondria, and contribute to CD8^+^ T cell exhaustion (*8*). These defects exacerbate metabolic stress and further impair the functionality of exhausted T cells (*8*). However, the molecular mechanisms underlying the dysregulation of mitophagy during T cell exhaustion are not well-defined.

One of the key pathways for mitophagy regulation involves phosphatase and tensin homologue (PTEN)–induced putative kinase 1 (PINK1) and the E3 ubiquitin ligase, Parkin (*22*, *23*). Under mitochondrial stress, PINK1 accumulates on the outer mitochondrial membrane (OMM) of damaged mitochondria, where it recruits and activates Parkin (*23*, *24*). This process leads to the ubiquitination of mitochondrial proteins, which promotes mitophagy and subsequent degradation of dysfunctional mitochondria (*22*, *25*–*27*). Notably, Parkin deficiency has been associated with impaired mitochondrial clearance in CD8^+^ T cells (*8*, *20*, *28*). Although the PINK1/Parkin pathway is well characterized, the role of mitochondrial deubiquitination, notably by ubiquitin-specific protease 30 (USP30), is less explored. USP30 counteracts Parkin-mediated mitophagy by deubiquitylating OMM proteins (*29*–*31*). Emerging evidence suggests that USP30-mediated suppression of mitophagy contributes to the accumulation of damaged mitochondria (*31*–*33*). Deletion or pharmacological inhibition of USP30 could enhance mitophagy and improve mitochondrial function (*29*, *34*), suggesting a specific strategy to selectively promote mitophagy. However, the extent to which USP30 contributes to impaired mitophagy in exhausted CD8^+^ T cells, as well as the therapeutic potential of targeting USP30, remains to be fully determined. Further investigation is warranted to understand USP30’s impact on CD8^+^ T cell mitophagy and to assess its therapeutic potential.

In this study, we identify USP30 as a T cell receptor (TCR)/nuclear factor of activated T cell 1 (NFATc1)–induced negative regulator of mitophagy that promotes CD8^+^ T cell exhaustion. Inhibiting USP30 restores mitophagy and mitochondrial fitness, rejuvenates effector function, and enhances antitumor immunity. These findings highlight mitophagy enhancement via USP30 inhibition as a promising immunotherapeutic strategy to combat T cell exhaustion in cancer.

## RESULTS

### Mitophagy is suppressed during T cell exhaustion

**Fig. 1.**
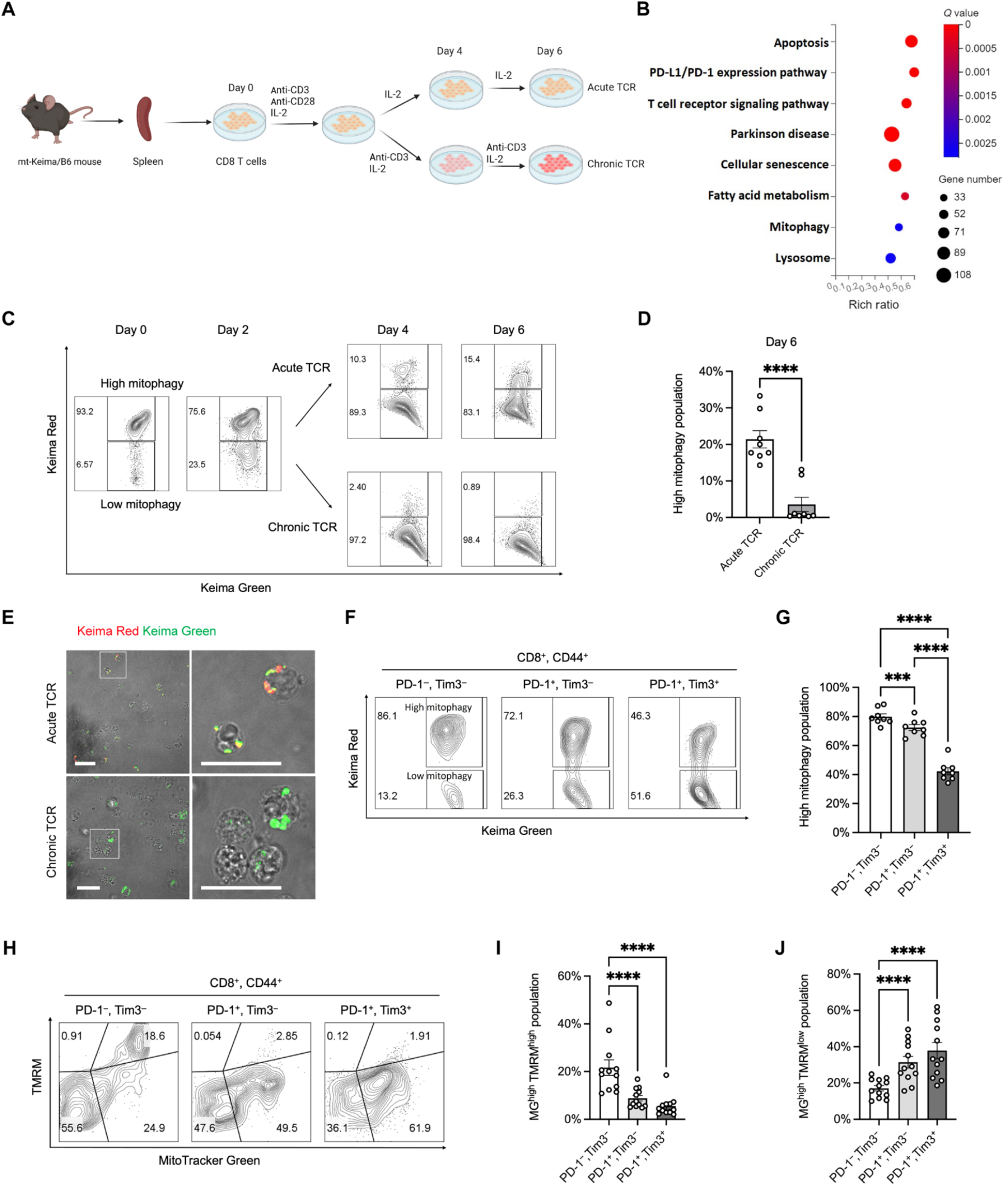
Decline of mitophagy in exhausted CD8^+^ T cells. (**A**) Experimental schematic of chronic and acute stimulation of CD8^+^ T cells. (**B**) Pathway enrichment analysis of differentially expressed genes showing down-regulated signatures related to mitophagy in exhausted CD8^+^ T cells. (**C**) Representative flow cytometry analysis of mitophagy in chronically and acutely stimulated CD8^+^ T cells from the mt-Keima reporter mouse. (**D**) Quantification of high mitophagy populations on day 6 in acutely and chronically stimulated CD8^+^ T cells. (**E**) Representative images of mt-Keima fluorescence in CD8^+^ T cells under acute and chronic TCR stimulation. Scale bars, 20 μm. (**F**) Representative flow cytometry analysis of mitophagy and quantification of high mitophagy population (**G**) in PD-1^−^Tim3^−^, PD-1^+^Tim3^−^, and PD-1^+^Tim3^+^ subsets. (**H**) Representative flow cytometry analysis of mitochondrial mass and membrane potential [(MitoTracker Green (MG) and tetramethylrhodamine methyl ester (TMRM)] and quantification of MG^high^TMRM^high^ population (**I**) and MG^high^TMRM^low^ population (**J**) in PD-1^−^Tim3^−^, PD-1^+^Tim3^−^, and PD-1^+^Tim3^+^ subsets. (C to I) Numbers in flow cytometry plots represent the percentage of the parent population within each gate, with gating based on CD44^+^CD8^+^ live singlets. Data represent means ± SEM, and the points correspond to the number of samples. ***P < 0.001; ****P < 0.0001.

Mitochondrial dysfunction and impaired metabolism are hallmarks of terminally exhausted T cells (*9*). To investigate how changes in mitochondrial function and cellular metabolism contribute to the development of T cell exhaustion, we used a well-established in vitro model of CD8^+^ T cell exhaustion (*6*). Activated T cells were expanded in the absence (“acute”) or presence (“chronic”) of persistent antigenic stimulation via anti-CD3–mediated TCR activation. Cells were passaged every 2 days with or without persistent stimulation, and both acutely and chronically stimulated T cells were briefly restimulated for 6 hours before collection (Fig. 1A). Multiple lines of evidence indicate that inhibitory receptors, such as programmed cell death protein 1 (PD-1) and Tim3, positively correlate with CD8^+^ T cell exhaustion and are highly expressed on terminal exhausted CD8^+^ T cells (*8*, *35*, *36*). Flow cytometry analysis demonstrated that CD8^+^ T cells in the chronic stimulation model began exhibiting exhaustion by day 4, as marked by increased PD-1 and Tim3 expression. Prolonged chronic stimulation further led to an elevated proportion of PD-1^+^Tim3^+^ terminal exhausted CD8^+^ T cells (fig. S1, A and B).

Because chronic stimulation in vitro was sufficient to activate a transcriptional signature associated with T cell exhaustion, we next examined global transcriptional changes induced by acute versus chronic stimulation. Consist with the signature of exhausted CD8^+^ T cells, genes related to the PD-1/programmed death-ligand 1 (PD-L1) signaling axis and TCR pathways were significantly enriched in chronically stimulated CD8^+^ T cells (Fig. 1B). Notably, genes associated with mitophagy and lysosome were significantly reduced in CD8^+^ T cells subjected to chronic stimulation (Fig. 1B). Mitophagy is a critical mechanism for maintaining mitochondrial quality control (*37*). To directly assess dynamic changes of mitophagy during chronic CD8^+^ T cell stimulation, we used the pH-sensitive mitochondria-targeted Keima (mt-Keima) fluorescent reporter mouse. The mt-Keima probe enables rapid and accurate determination of whether mt-Keima–tagged mitochondria are at the physiological pH (pH 8.0, green) or within autophagosomes that have fused with lysosomes for mitophagic degradation (pH 4.5, red) (*38*). This differentiation can be effectively detected using flow cytometry. While more than 90% of native CD8^+^ T cells displayed high levels of mitophagy, TCR stimulation led to a significant reduction in mitophagy over a 48-hour period (Fig. 1C). Additionally, the mitophagy^high^ population progressively declined with prolonged in vitro stimulation (Fig. 1C). Notably, after day 4, chronically stimulated cells exhibited much lower mitophagy activity, with only ~1% of cells showing mitophagy on day 6, compared to more than 10% in acutely stimulated cells (Fig. 1, C and D), indicating that mitophagy is inhibited in exhausted CD8^+^ T cells. We further analyzed mitophagy using laser-scanning confocal microscopy and confirmed that chronic TCR stimulation resulted in decreased overall red mt-Keima fluorescence compared to those under acute stimulation, consistent with suppressed mitophagy (Fig. 1E). To validate these findings using mt-Keima–independent approaches, we assessed autophagic flux by analyzing lipidation of microtubule-associated protein 1 light chain 3 (LC3; LC3-II/LC3-I ratio) (*39*) in CD8^+^ T cells following acute or chronic stimulation. Immunoblotting revealed a reduced LC3-I to LC3-II conversion under chronic stimulation, along with elevated levels of the mitophagy adaptor p62 (*40*), both indicative of impaired autophagic activity (fig. S1C). We extended these findings in tumor-bearing mt-Keima mice by analyzing tumor-infiltrating CD8^+^ T cells across different stages of exhaustion, as defined by the expression dynamics of PD-1 and Tim3 expression (*41*–*43*). Flow cytometry results demonstrated that the nonexhausted PD-1^−^Tim3^−^ subset exhibited the highest proportion of the mitophagy^high^ population, while the terminally exhausted PD-1^+^Tim3^+^ CD8^+^ T cells showed the lowest proportion (Fig. 1, F and G). These findings suggest that CD8^+^ T cells experience impaired mitophagy dynamic as the exhaustion progresses.

A decline in mitophagic flux has been widely proposed to contribute to the accumulation of damaged mitochondria in T cells (*43*). To investigate whether mitochondrial dysfunction is associated with impaired mitophagy in exhausted CD8^+^ T cells, we assessed mitochondrial fitness using MitoTracker Green (MG), which labels mitochondria mass, and tetramethylrhodamine methyl ester (TMRM), a cationic dye used to measure MMP. Consistent with previous findings (*8*), we observed depolarized mitochondria characterized by increased MG staining and reduced TMRM signal, while functional mitochondria displayed high levels of both MG and TMRM staining. In line with impaired mitophagy, the proportion of cells harboring functional mitochondria (MG^high^TMRM^high^) progressively declined, reaching its lowest level in terminally exhausted PD-1^+^Tim3^+^ CD8^+^ T cells (Fig. 1, H to J). Concurrently, this terminally exhausted subset exhibited a significant accumulation of depolarized mitochondria (MG^high^TMRM^low^) (Fig. 1, H to J). Moreover, CD8^+^ T cells with dysfunctional mitochondria showed elevated expression of inhibitory markers and a higher proportion of terminally exhausted CD8^+^ T cells (fig. S1, D to G). These findings suggest that disruption of mitophagy may contribute to mitochondrial dysfunction in exhausted CD8^+^ T cells.

### Up-regulation of USP30 contributed to mitophagy inhibition in exhausted CD8^+^ T cells

**Fig. 2.**
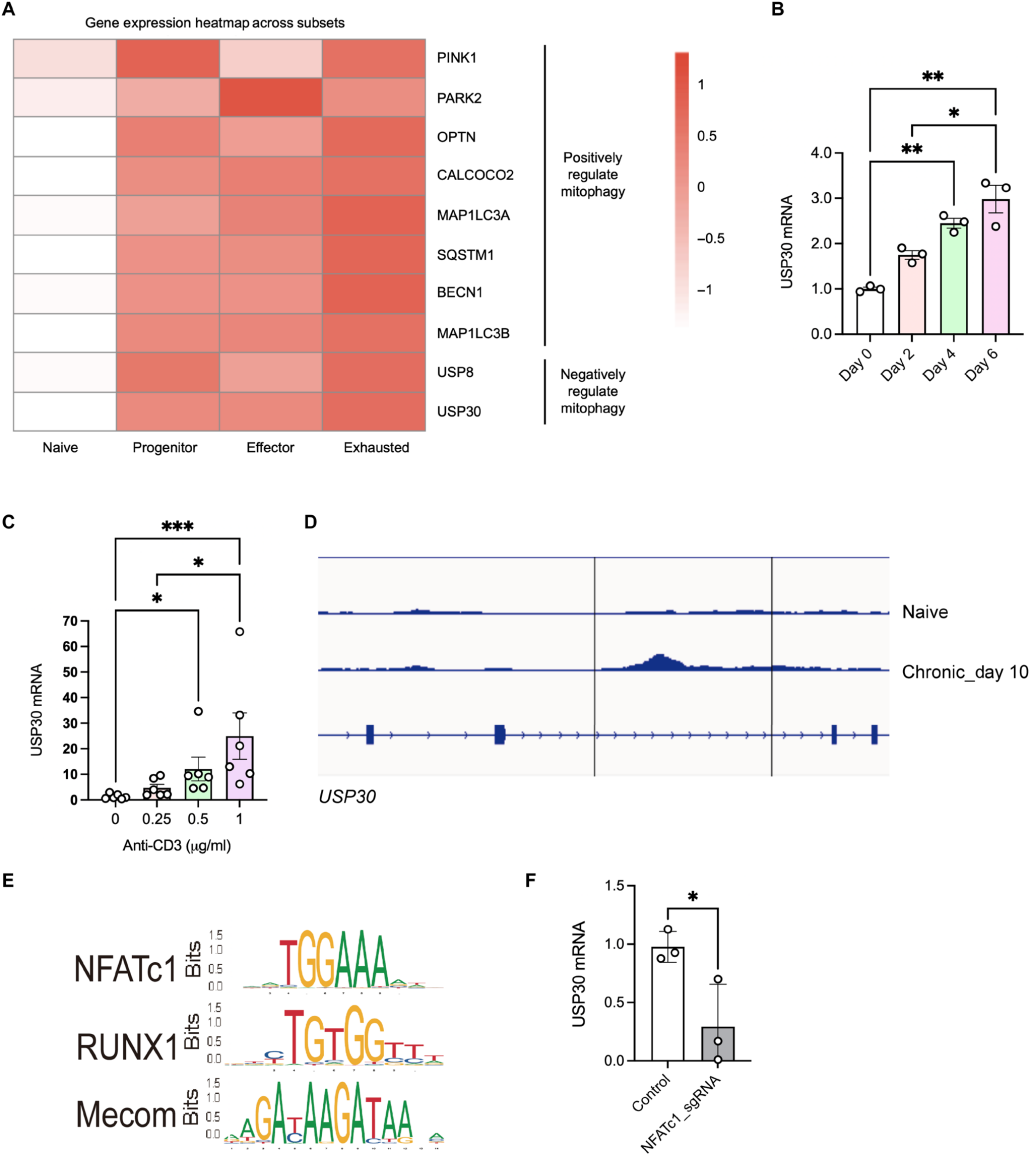
TCR/NFATc1-mediated up-regulation of USP30 inhibits mitophagy in exhausted CD8^+^ T cells. (**A**) Heatmap of mitophagy-related gene expression in naive (CD44^−^), progenitor (Ly108^+^), effector (CX3CR1^+^), and exhausted (Tim3^+^) colon tumor-infiltrated CD8^+^ T cells. Data from recently published single-cell analyses of human CRC datasets are available via the Single Cell Portal (SCP259). (**B**) Quantitative polymerase chain reaction (qPCR) analysis of USP30 expression in CD8^+^ T cells subjected to chronic TCR stimulation for 0, 2, 4, and 6 days. Significance was determined using one-way analysis of variance (ANOVA). *P < 0.05; **P < 0.01. (**C**) qPCR analysis of *USP30* expression in CD8^+^ T cells stimulated with anti-CD3 antibody at doses of 0, 0.25, 0.5, and 1 μg/ml. Significance was determined using one-way ANOVA. (**D**) Transposase-accessible chromatin sequencing (ATAC-seq) signal tracks for chronically stimulated CD8^+^ T cells at day 0 (top) and day 10 (bottom); chr5:114,100,000-114,220,000 exon of *USP30* locus showed in the peak. Data from the public ATAC-seq datasets (GSE203593). (**E**) The motif analysis was performed on published ATAC-seq data (GSE203593) from activated CD8^+^ T cells. The consensus motif enriched within the regulatory region of *USP30* loci. (**F**) qPCR analysis of *USP30* expression in CD8^+^ T cells after CRISPR-mediated *NFATc1* KO. Data represent means ± SEM, and the points correspond to the number of samples. *P < 0.05; ***P < 0.001.

To further elucidate how mitophagy becomes impaired during T cell exhaustion, we conducted a transcriptome analysis focusing on genes that regulate mitophagy, both positively and negatively. In contrast to our findings, which show that exhausted T cells exhibit lower mitophagy, reanalysis of single-cell RNA sequencing (RNA-seq) data from recently published human colorectal cancer (CRC) datasets (SCP259) (*44*) revealed a general up-regulation of genes that promote mitophagy in exhausted CD8^+^ T cell subsets, compared to naïve T cells (Fig. 2A). Notable up-regulated genes include *BECN1*, *PINK1*, and *PARK2* and mitophagy receptors such as *OPTN*, *CALCOCO2*, and *SQSTM1* (Fig. 2A). The expression levels of *USP8* and *USP30*, negative regulators of mitophagy (*31*), were increased in the progenitor, effector, and exhausted CD8^+^ T cell subsets, compared to those in naïve T cells (Fig. 2A). In particular, *USP30* exhibited the highest expression levels specifically in exhausted CD8^+^ T cell subsets (Fig. 2A). This suggests that T cell exhaustion may, at least, in part, suppress mitophagy through the up-regulation of *USP30*. Supporting this hypothesis, we observed a progressive increase in *USP30* transcript levels over a 6-day chronic stimulation period in CD8^+^ T cells (Fig. 2B). To validate this observation in a defined antigen-specific model, we used CD8^+^ T cells from Pmel-1 transgenic mice, which specifically recognize GP100 and subjected them to chronic stimulation with the GP100 peptide (fig. S2A). Prolonged exposure to GP100 led to an increased frequency of PD-1^+^Tim3^+^ terminally exhausted Pmel-1 CD8^+^ T cells (fig. S2B). In line with the above observation, chronically stimulated Pmel-1 cells exhibited higher *USP30* expression compared to acutely stimulated counterparts (fig. S2C). These results indicate that *USP30* up-regulation is a conserved feature of CD8^+^ T cell exhaustion across both polyclonal and antigen-specific settings and may contribute functionally to the suppression of mitophagy in exhausted CD8^+^ T cells.

### *USP30* expression is transcriptionally regulated by TCR-induced activation of NFATc1

We next investigated the mechanism of *USP30* up-regulation in CD8^+^ T cells. Given that TCR signaling is a well-established driver of T cell exhaustion, we hypothesized that TCR activation might directly regulate *USP30* expression. As such, we treated mouse CD8^+^ T cells with varying concentrations of anti-CD3 antibody to simulate different levels of TCR stimulation. We demonstrated a dose-dependent increase in *USP30* expression, with higher concentrations of anti-CD3 antibody resulting in greater *USP30* up-regulation (Fig. 2C). To investigate the transcriptional regulation of *USP30* during exhaustion, we reanalyzed the transposase-accessible chromatin sequencing (ATAC-seq) datasets from chronic TCR stimulated CD8 T cells (*8*, *45*). We found that genomic regions surrounding the *USP30* locus exhibited increased chromatin accessibility (Fig. 2D). To identify transcription factors (TFs) that could be involved in regulating *USP30* expression, we performed TF binding motif enrichment analysis around this locus. This analysis revealed multiple TFs with enriched motifs, including NFATc1, a key TF downstream of TCR signaling that plays a pivotal role in T cell activation and function (Fig. 2E). To further confirm the role of NFATc1, we used a CRISPR-Cas9–mediated approach to knockout (KO) NFATc1 in CD8^+^ T cells, followed by TCR activation. Notably, the loss of NFATc1 led to a significant reduction in *USP30* expression compared to control cells (Fig. 2F). Therefore, USP30 expression, at least partially, is transcriptionally regulated by NFATc1, in response to TCR signaling, and plays a role in the progression of CD8^+^ T cell exhaustion.

### USP30 deficiency improves effector function in exhausted CD8^+^ T cells

To investigate the functional role of USP30 in CD8^+^ T cell responses, we used a CRISPR-Cas9–based KO strategy to delete USP30 in antigen-specific Pmel-1 CD8^+^ T cells. Splenocytes from Pmel-1 transgenic mice were stimulated with GP100 peptide and interleukin-2 (IL-2) for 24 hours and then electroporated with Cas9 ribonucleoprotein complexes containing either a nontargeting control or a USP30-specific guide RNA. We confirmed efficient USP30 deletion by reverse transcription–quantitative polymerase chain reaction (qPCR) (fig. S3A). Compared to control cells, USP30-deficient CD8^+^ T cells exhibited reduced the expression of PD-1 and Tim3 following prolonged GP100 stimulation (fig. S3, B and C). Notably, the deletion of USP30 significantly elevated the maximal respiration rate (fig. S3D), indicative of functional mitochondria. Together, these findings demonstrate that USP30 deletion mitigates T cell exhaustion and preserves mitochondrial integrity, underscoring USP30 as a promising therapeutic target for enhancing antitumor CD8^+^ T cell responses.

**Fig. 3.**
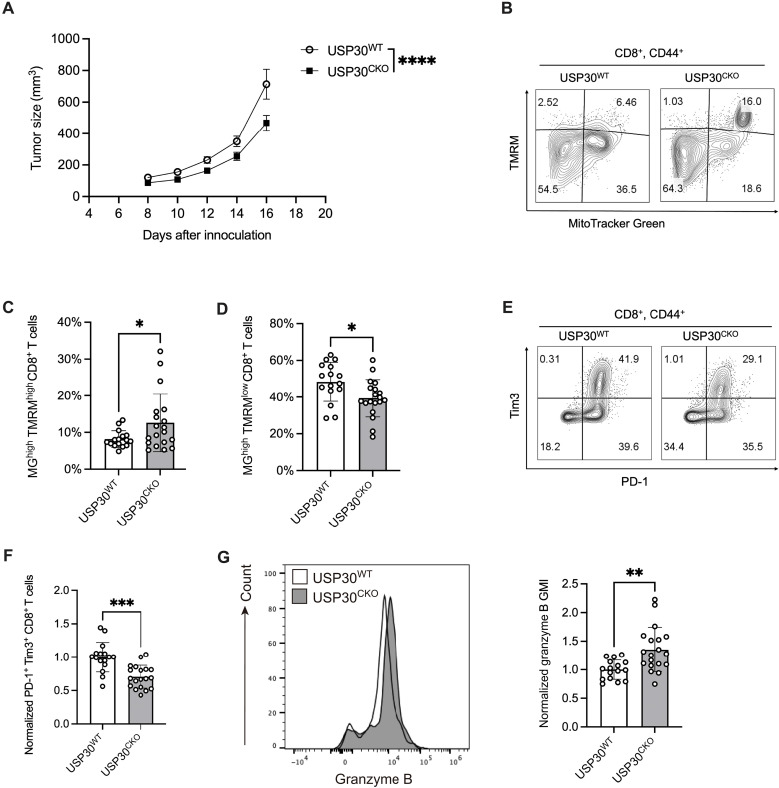
USP30 deletion enhances effector function in CD8^+^ T cells. (**A**) USP30^WT^ and USP30^CKO^ mice were subcutaneously inoculated with MC38 tumor cells. Tumor growth kinetics in USP30^WT^ and USP30^CKO^ mice over 16 days post–MC38 tumor cell inoculation, with tumor sizes measured every other day; significance was calculated by two-way ANOVA. ****P < 0.0001. (**B**) Representative flow cytometry analysis of MMP (TMRM) and mitochondrial mass (MG), (**C**) quantification of the MG^+^TMRM^+^ population, and (**D**) MG^+^TMRM^−^ population in tumor-infiltrating CD8^+^CD44^+^ T cells from USP30^WT^ and USP30^CKO^ mice. Significance was calculated by unpaired *t* test. *P < 0.05. (**E**) Representative flow cytometry plots showing PD-1 and Tim3 expression in tumor-infiltrating CD8^+^CD44^+^ T cells from USP30^WT^ and USP30^CKO^ mice. (**F**) Flow cytometry analysis of the percentage of PD-1^+^Tim3^+^CD8^+^ tumor-infiltrating T cells in USP30^WT^ and USP30^CKO^ mice; significance was calculated by unpaired *t* test. ***P < 0.001. (**G**) Normalized flow cytometry analysis of granzyme B expression. GMI, geometric mean intensity. in tumor-infiltrating CD8^+^ T cells from USP30^WT^ and USP30^CKO^ mice; significance was calculated by unpaired *t* test. **P < 0.01. Data represent means ± SEM, and the points correspond to the number of samples.

The particular role of USP30 in CD8^+^ T cell–mediated antitumor immunity has not been described. To determine whether USP30-mediated suppression of mitophagy affects CD8^+^ T cell function, we generated mice with T cell–specific deletion of USP30 by crossing USP30^flox/flox^ mice with transgenic mice expressing Cre recombinase under the control of the *Lck* (lymphocyte protein tyrosine kinase) promoter (*46*, *47*) (USP30^CKO^). We confirmed the ablation of *USP30* expression in CD8^+^ T cells (fig. S4A). These USP30-deficient CD8^+^ T cells exhibited elevated autophagic flux, indicated by an increased LC3-II/I ratio and reduced expression of the mitophagy receptors p62 and OPTN (fig. S4B). The USP30^CKO^ mice were phenotypically normal and displayed unaltered T cell profiles (fig. S4C) at homeostasis. To investigate the role of USP30 in tumor immunity, we inoculated MC38 murine colon adenocarcinoma cells into both USP30^CKO^ and control USP30^WT^ (USP30^flox/flox^; Lck^Cre−^) mice. Notably, tumor growth was significantly reduced in USP30^CKO^ mice, suggesting that USP30 deletion in CD8^+^ T cells enhanced antitumor efficiency (Fig. 3A). Analysis of tumor-infiltrating lymphocytes (TILs) revealed that USP30 deletion significantly increased the MG^+^TMRM^+^ CD8^+^ T cell population, indicating improved mitochondrial fitness (Fig. 3, B to D). Furthermore, USP30^CKO^ mice exhibited a notable reduction in the proportion of terminally exhausted PD-1^+^Tim3^+^ CD8^+^ T cells (Fig. 3, E and F) accompanied by a significant increase in granzyme B expression (Fig. 3G), reflecting enhanced cytotoxic function of CD8^+^ T cells within the TME. In contrast, the analysis of CD4^+^ and CD8^+^ T cells from the draining lymph nodes showed that USP30 deletion had no significant impact on T cells outside the TME (fig. S4, D and E). Collectively, these data indicate that USP30 deletion improves mitochondrial fitness and restores effector function in exhausted CD8^+^ T cells. This suggests that targeting USP30-regulated mitophagy could represent a promising therapeutic strategy to reinvigorate exhausted CD8^+^ T cells, offering potential benefits in the treatment of cancer.

### Pharmacological inhibition of USP30 restores effector function in exhausted CD8^+^ T cells

**Fig. 4.**
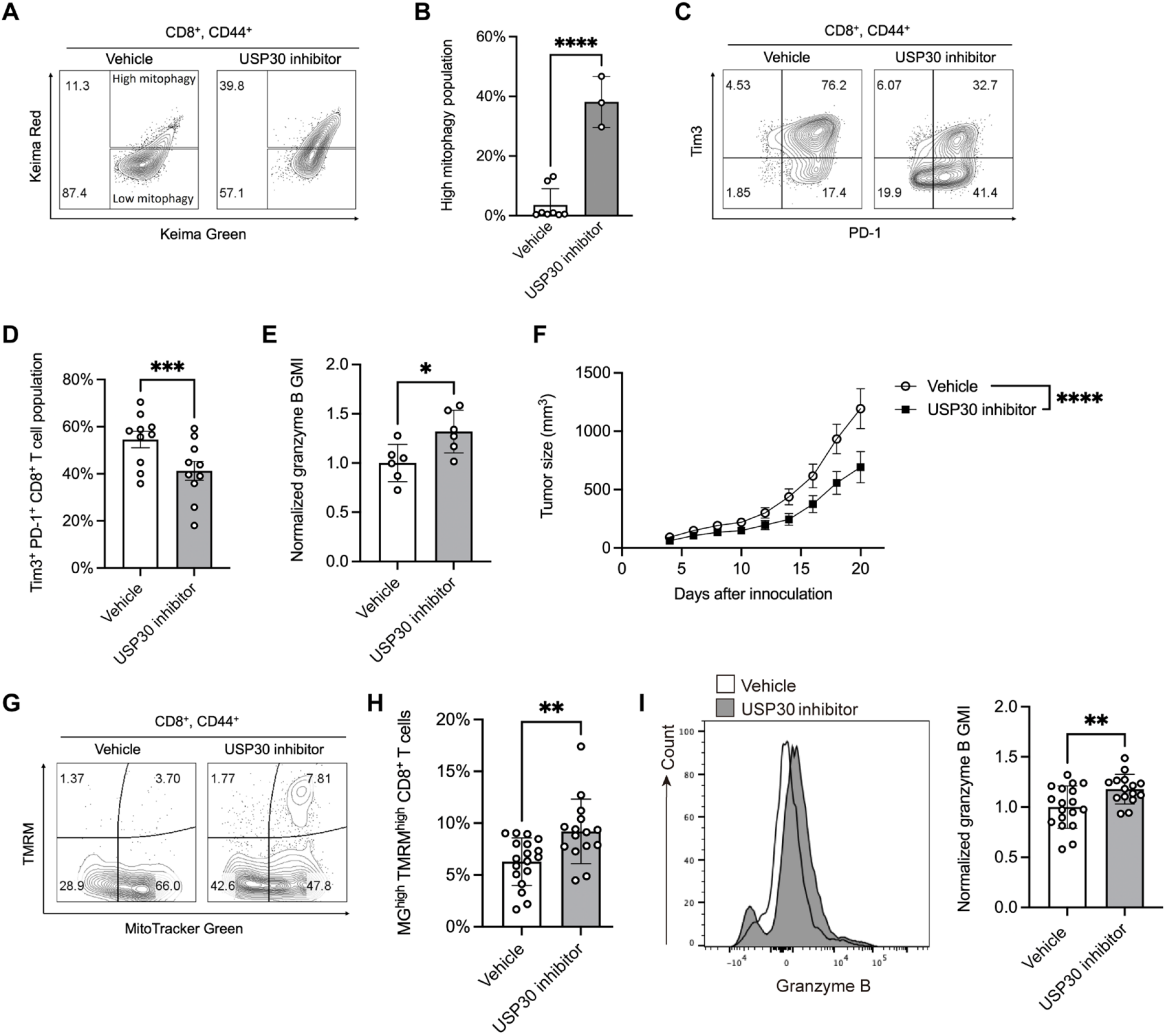
Inhibition of USP30 restores effector functions in exhausted CD8^+^ T cells. (**A**) CD8^+^ T cells were isolated from the mt-Keima mice and subjected to chronic TCR stimulation. Two days after initial stimulation, cells were treated with either vehicle or the USP30 inhibitor. On day 6 poststimulation, mitophagy levels were assessed by flow cytometry; a representative plot is shown. (**B**) Quantification of the mitophagy^high^ population in CD8^+^CD44^+^ T cells treated with vehicle or USP30 inhibitor. Significance was calculated by unpaired *t* test. ****P < 0.0001. (**C**) CD8^+^ T cells from C57BL/6 mice were subjected to the same chronic TCR stimulation and USP30 inhibitor treatment. The expression of PD-1 and Tim3 on CD8^+^CD44^+^ T cells was assessed by flow cytometry. (**D**) Quantification of Tim3^+^PD-1^+^CD8^+^ T cell populations. Significance was determined by unpaired *t* test. ***P < 0.001. (**E**) Normalized flow cytometry analysis of Granzyme B expression (GMI) in CD8^+^ T cells treated with vehicle or USP30 inhibitor. Significance was determined by unpaired *t* test. *P < 0.05. (**F**) C57BL/6 mice were subcutaneously inoculated with MC38 tumor cells. Beginning on day 6 postinoculation, mice received either vehicle or the USP30 inhibitor daily for 8 days. Tumor growth was monitored over a 20-day period. Tumor sizes were measured every other day; significance was calculated by two-way ANOVA. ****P < 0.0001. (**G**) Representative flow cytometry analysis of TMRM and MG, and (**H**) quantification of the MG^+^TMRM^+^ population in tumor-infiltrating CD8^+^CD44^+^ T cells from mice treated with vehicle or USP30 inhibitor; significance was calculated by unpaired *t* test. **P < 0.01. (**I**) Normalized flow cytometry analysis of granzyme B expression (GMI) in tumor-infiltrating CD8^+^ T cells from mice treated with vehicle or USP30 inhibitor; significance was calculated by unpaired *t* test. **P < 0.01. Data represent means ± SEM, and the points correspond to the number of samples.

We next explored whether pharmacological inhibition of USP30 could promote mitophagy and improve the function of exhausted CD8^+^ T cells. We used ST-539, a USP30-specific inhibitor known to stimulate mitophagy (*32*). We tested the ability of ST-539 to reverse mitophagy impairment in exhausted CD8^+^ T cells in vitro in response to polyclonal T cell activation. ST-539 treatment led to over a threefold increase in CD8^+^ T cell mitophagy (Fig. 4, A and B) and markedly enhanced mitochondrial efficiency (fig. S5, A to C). Furthermore, ST-539 significantly reduced the proportion of terminally exhausted PD-1^+^Tim3^+^ CD8^+^ T cells while also boosting the expression of the effector molecular granzyme B, a key mediator of CD8^+^ T cell cytotoxicity (Fig. 4, C to E). Comparable improvements in mitochondrial fitness (fig. S6, A and B), reduced exhaustion marker expression (fig. S6, C to E), and enhanced effector function (fig. S6F) were also observed in the Pmel-1 model following ST-539 treatment. These findings indicate that ST-539 effectively augments mitophagy and limits CD8^+^ T cell exhaustion.

To further assess the antitumor efficacy of ST-539 in vivo, MC38 tumor cells were subcutaneously inoculated into C57BL/6 mice, followed by oral administration of ST-539 every other day. The tumor growth curve showed a significant reduction in the ST-539–treated group compared to that in controls, underscoring its potent antitumor activity (Fig. 4F). Notably, ST-539 demonstrated minimal direct cytotoxicity against MC38 tumor cells (fig. S7A) and had mild impact on T cell expansion (fig. S7B). We then analyzed TILs to evaluate mitochondrial fitness. Consistent with observations in USP30^CKO^ mice, ST-539 treatment resulted in a twofold increase in the MG^+^TMRM^+^ CD8^+^ T cell population (Fig. 4, G and H), suggesting enhanced mitochondrial fitness. ST-539 also significantly increased granzyme B levels in CD8^+^ TILs from MC38 tumors (Fig. 4I), further indicating improved effector functionality. Subsequent investigations into tumor-associated CD4^+^ T cells revealed that ST-539 treatment had no significant impact on their mitochondrial activity (fig. S7C). Similarly, the frequencies of regulatory T cells, macrophages, myeloid cells, and natural killer (NK) cells remained unchanged (fig. S7, D to G), suggesting that the effects of USP30 inhibition are specific to CD8^+^ T cells. Collectively, these findings demonstrate that pharmacological inhibition of USP30 with ST-539 alleviates mitochondrial dysfunction and enhances antitumor immunity by protecting CD8^+^ T cells from exhaustion.

### USP30 inhibitor ST-539 improves mitochondrial health and mitigates exhaustion in chronically stimulated human CD8^+^ T cells

**Fig. 5.**
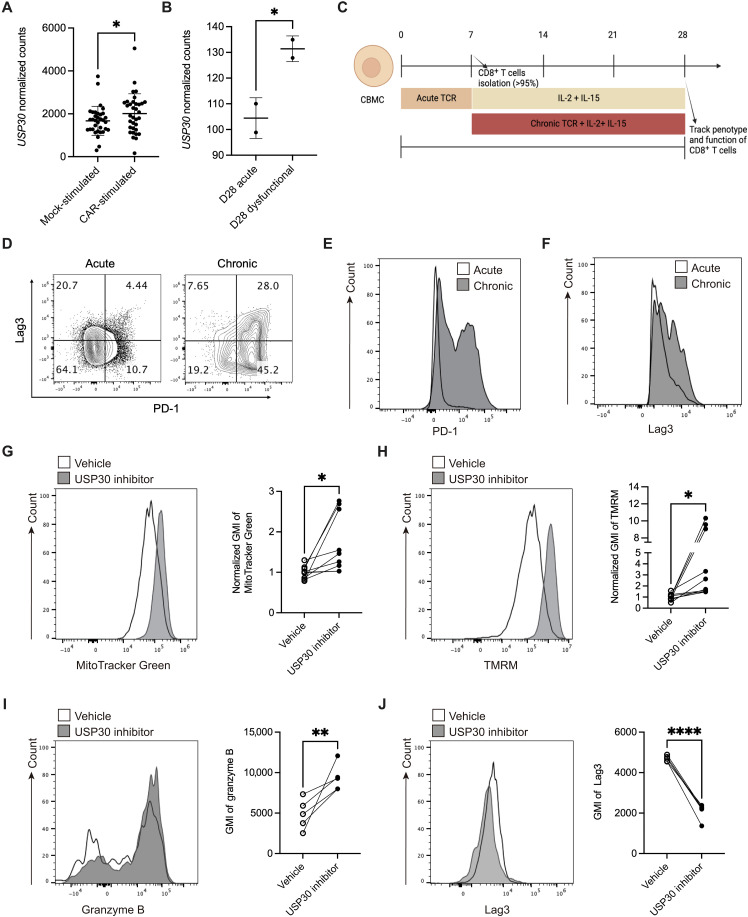
Inhibition of USP30 mitigates exhaustion in human CD8^+^ T cells. (**A**) The bulk sequencing dataset from CD19 CAR T cells received mock or CAR-specific stimulation (*48*). The gene expression level of *USP30* was compared between these two groups. (**B**) The transcript level of *USP30* was evaluated in dysfunctional and acute stimulated CD8 T cells derived from cord blood mononuclear cell (CBMC) model of T cell exhaustion (*49*). (**C**) In vitro model of human CD8^+^ T cell exhaustion using CBMCs. (**D** to **F**) Representative flow cytometry plots of PD-1 and Lag3 expression in CD8^+^ T cells under acute or chronic antigen stimulation. (**G** and **H**) Representative flow cytometry analysis and quantification of mitochondrial mass (MG) (G) and MMP (TMRM) (H) in CD8^+^ T cells from CBMCs treated with vehicle or USP30 inhibitor. Significance was determined by unpaired *t* test. *P < 0.05. (**I** and **J**) Normalized flow cytometry analysis of granzyme B (I) and Lag3 (J) expression (GMI) in CD8^+^ T cells from CBMCs treated with vehicle or USP30 inhibitor. Significance was determined by unpaired *t* test. **P < 0.01; ****P < 0.0001.

To explore the translational value of USP30 inhibition, we reanalyzed published RNA-seq datasets from CD19 chimeric antigen receptor (CAR) T cells and found that CAR-specific stimulation significantly up-regulated USP30 compared to mock stimulation (Fig. 5A) (*48*). Similarly, in an in vitro model of human T cell exhaustion, bioinformatic analysis of a published dataset (*49*) confirmed that USP30 expression is significantly elevated in dysfunctional CD8^+^ T cells compared to that in acutely stimulated counterparts (Fig. 5B) (*49*). To assess the functional consequences of USP30 inhibition, we used a cord blood mononuclear cell (CBMC) model of CD8^+^ T cell exhaustion (Fig. 5C) (*49*). CBMCs were stimulated under acute and chronic conditions to induce distinct T cell states (Fig. 5C). Flow cytometry analysis revealed that prolonged chronic stimulation significantly increased the proportion of PD-1^+^Lag3^+^ exhausted CD8^+^ T cells, along with elevated expression levels of PD-1 and Lag3 (Fig. 5, D to F), which confirm terminally dysfunctional state. Notably, USP30 inhibition enhanced mitochondrial efficiency in chronically stimulated human CD8^+^ T cells (Fig. 5, G and H) and boosted granzyme B expression (Fig. 5I), reflecting improved cytotoxic potential. Consistently, Lag3 protein levels were significantly reduced following ST-539 treatment (Fig. 5J). Together, these findings suggest that USP30 inhibition promotes mitochondrial health and effector function in chronically stimulated human CD8^+^ T cells.

## DISCUSSION

One of the key features of T cell exhaustion is mitochondrial dysfunction, which substantially impairs their effector function, contributing to a reduced immune response during chronic infections and cancer (*6*, *8*). While strategies such as enhancing mitochondrial biogenesis have been explored, these approaches fail to address the accumulation of damaged mitochondria in T cells (*50*, *51*). As such, considerable progress has been made in understanding the role of mitophagy as a mechanism of mitochondrial quality control in T cells (*52*). Central to this process are PINK1 and Parkin, which mediate the ubiquitination and removal of damaged mitochondria (*53*, *54*). However, the role of mitochondrial deubiquitination in T cell mitophagy has remained largely unexplored (*8*, *20*, *55*). Here, we demonstrate that mitophagy declines in CD8^+^ T cells upon TCR activation and further exacerbated as T cells progress toward exhaustion (Fig. 1, C and D). Concurrently, USP30, a mitochondrial deubiquitinase, is up-regulated in exhausted CD8^+^ T cells and contributes to mitophagy suppression (Fig. 2, A and B). We identified NFATc1, a TCR-activated TF, as a key regulator of USP30 expression (*56*, *57*), establishing a direct link between TCR signaling and mitochondrial quality control. These findings reveal a previously unrecognized mechanism by which chronic antigen stimulation drives mitochondrial dysfunction and T cell exhaustion through transcriptional up-regulation of *USP30*.

Although mitophagy has been implicated in T cell homeostasis, the specific regulatory mechanisms in the context of exhaustion remain incompletely understood (*8*). The functional significance of mitophagy in maintaining T cell mitochondrial homeostasis has been evaluated through various loss-of-function genetic approaches targeting various mitophagy regulators, such as Parkin and PINK1 (*20*, *58*–*60*). While recent studies suggested that USP30 may influence mitochondrial translation and in vitro cytotoxic function (*61*, *62*), its role in T cell exhaustion and in vivo antitumor immunity had not been defined. Furthermore, the USP30 whole-body KO mice (*62*), which exhibit broad systemic deletion, may display phenotypes distinct from those observed in our T cell–specific KO model. We selectively delete USP30 in T cells and investigate its T cell–intrinsic role in vivo. Consistent with the previous study (*62*), we confirmed the ablation of USP30 specifically in T lymphocyte cells did not affect baseline T cell development or homeostasis. However, we found that USP30 deletion markedly improved mitochondrial fitness and partially restored effector function in exhausted CD8^+^ T cells in a colon cancer setting. Together, these findings suggest that USP30 may have tissue-specific functions and that its role in T cells may be various depending on in vitro activation and in vivo exhaustion.

Our study provides several lines of evidence indicating that genetic deletion or pharmacological inhibition of USP30 improves mitochondrial fitness, which is essential for T cell function. While we focused on mitophagy, USP30 may also regulate other aspects of mitochondrial homeostasis (*31*, *61*, *63*). Given that T cell dysfunction is a major limitation to immune checkpoint blockade, targeting mitochondrial dysfunction via USP30 inhibition may offer a complementary approach. Previous study indicates the possibility of activating mitophagy by modulating the sirtuin pathway using nicotinamide riboside (NR) or nicotinamide mononucleotide treatment (*8*). NR treatment markedly reduces the accumulation of dysfunctional mitochondria and improves the antitumor effector function (*8*). Consequently, dietary supplementation with NR elicited additive antitumor responses in combination with anti–PD-1 therapy (*8*). One important consideration is the potential for excessive mitophagy upon USP30 inhibition, which could lead to bioenergetic insufficiency due to mitochondrial depletion. This highlights the need for optimizing dose and treatment timing based on tumor context and T cell metabolic demand. Fine-tuning the dose and treatment schedule could potentially modulate the impact of USP30 inhibition, maximizing its therapeutic benefit while minimizing off-target effects. This dose-dependent effect could also vary across tumor types, as different cancers may present distinct mitochondrial and metabolic challenges for infiltrating CD8^+^ T cells. Future studies will need to explore the long-term impact of USP30 inhibition, including any possible unintended consequences of overstimulating mitophagy, such as excessive removal of mitochondria, which could impair overall T cell bioenergetics. In addition, a detailed analysis of the metabolic and transcriptional landscapes in human CD8^+^ T cells following USP30 inhibition is crucial to understanding its broader immunological effects and evaluating its safety as a therapeutic approach. These studies are essential for advancing USP30 inhibition as a promising strategy to reverse T cell exhaustion and enhance antitumor immunity in clinical applications. In summary, our findings establish USP30 as a key regulator of mitophagy and mitochondrial fitness in CD8^+^ T cells. Targeting USP30 represents a promising, mitochondria-directed strategy to restore T cell function in exhausted states and may enhance the efficacy of immunotherapies in cancer.

## METHODS

### Cell culture

The MC38 cell line was obtained from the American Type Culture Collection and cultured in RPMI 1640 supplemented with 10% fetal bovine serum (FBS). Mouse CD8^+^ T cells were cultured in T cell medium containing RPMI 1640, 10% FBS, 2 mM l-glutamine, penicillin-streptomycin, nonessential amino acids, 1 mM sodium pyruvate, 5 mM Hepes buffer, and β-mercaptoethanol. The cultures were incubated in temperature-stable and partial-pressure-stable conditions at 37°C and 5% CO_2_.

### CD8^+^ T cell activation in vitro

CD8^+^ T cells were isolated from mice spleen cells using the EasySep Mouse CD8^+^ T Cell Isolation Kit (catalog no. 19753). CD8^+^ T cells were activated in T cell medium by adding the anti-CD3 antibody (3 μg/ml, coated on the 24-well plate) (catalog no. 100359), anti-CD28 antibody (1 μg/ml) (catalog no. 102116), and IL-2 (100 IU) for early 2 days. On day 2, for acute TCR stimulation, activated T cells were cultured in T cell medium adding IL-2 (100 IU/ml). For chronic TCR stimulation, activated CD8^+^ T cells were cultured in T cell medium in plates precoated with anti-CD3 antibody (3 μg/ml, 50 μl per well, coated at 4°C overnight). The medium was supplemented with recombinant IL-2 (100 IU/ml) throughout the culture period. After 2 days, cells were collected, washed, and transferred to freshly coated plates, where the same stimulation protocol was repeated (anti-CD3 coating and IL-2 supplementation). This process was continued for multiple stimulation cycles, as indicated in Fig. 1A. For all in vitro experiments, the concentration of T cells was kept under 1 million cells/ml. For Pmel-1 experiments, splenocytes were isolated from Pmel-1 mice and activated with GP100 peptide (1 μg/ml). In the acute stimulation condition, the peptide was present only during the initial 24 hours. For chronic stimulation, Pmel-1 CD8^+^ T cells were continuously maintained with GP100 peptides (200 ng/ml) continuously. In both conditions, cells were cultured in the presence of IL-2 (100 U/ml), with medium refreshed every other day.

### Mice

All experiments involving mice were conducted in accordance with the guidelines of the Institutional Animal Care and Use Committee at The Ohio State University. Both male and female mice were used in this study. We have previously described the mt-Keima mouse (*38*). The mt-Keima mouse line was backcrossed for over 10 generations and maintained on a C57BL/6J background. USP30^fl/fl^ mice were obtained from the European Mouse Mutant Archive [EM:09734, C57BL/6 N-Atm1Brd Usp30tm2a [the European Conditional Mouse Mutagenesis Program (EUCOMM) Hmgu/WtsiH] and crossed with the transgenic strain expresses Cre under the control of the lymphocyte protein tyrosine kinase promoter (LCK-cre) (*47*). Pmel-1 mice (strain no. 005023) were obtained from the Jackson Laboratory.

### Tumor collection and mechanical disruption

Single-cell suspensions of T cells were prepared by mechanically disrupting mouse spleens with the blunt end of a syringe plunger, followed by filtration through 70-μm strainers (Fisherbrand). For tumor single-cell suspensions, intact tumors were enzymatically treated by injecting a solution containing collagenase I (catalog no. C0130) and collagenase type IV (catalog no. C5138) in buffered RPMI medium. The tumors were incubated at 37°C for 60 min, mechanically disrupted with the blunt end of a syringe plunger and filtered through 70-μm strainers (Fisherbrand). Lysed tumor tissues were passed through strainers to obtain a cell mixture. Lymphocytes were subsequently isolated from the cell mixture using lymphocyte separation medium (Ficoll, catalog no. 17-5442-02).

### Flow cytometry

For cell surface marker staining, collected cells were washed with phosphate-buffered saline (PBS) and incubated with antibodies in fluorescence-activated cell sorting buffer (1% FBS, 2 mM EDTA, and 2 mM NaN_3_) for 30 min on ice. The antibodies used include Tim-3-APC Fire 750 (catalog no. 119721, clone RMT3-23), PD-1–phycoerythrin–cyanine 7 (PE–Cy7; catalog no. 135225, clone 29F.1A12), CD3–Pacific Blue (catalog no. 100214, clone 17A2), CD4-BUV737 (catalog no. 367-0041-82, clone GK1.5), CD44-BV785 (catalog no. 103049, clone IM7), CD8-BV510 (catalog no. 100752, clone 53-6.7), CD45-BV605 (catalog no. 103149, clone 30-F11), CD45–fluorescein isothiocyanate (FITC; catalog no. 103108, clone 30-F11), CD11b-BV510 (catalog no. 50402941, clone M1/70), F4/80-BV785 (catalog no. 123141, clone BM8), CD206-BV650 (catalog no. 141723, clone C068C2), MHCII-BUV395 (catalog no. 569244, clone M5/114.15.2), CD11c-AF700 (catalog no. 117326, clone N418), CD301-PE (catalog no. 146803, clone URA-1), CD80-BV605 (catalog no. 104729, clone 16-10A1), CD3–Pacific Blue (catalog no. 100214, clone 17A2), Ly6c-APC-fire810 (catalog no. 128056, clone HK1.4), Ly6G-AF647 (catalog no. 135225, clone 1A8), and NK1.1-BV570 (catalog no. 7453, clone PK136). For intracellular marker staining, after surface marker staining, cells were fixed using a fixation buffer and permeabilized overnight with True-Nuclear Fix. On the second day, permeabilized cells were incubated with antibodies in Perm buffer (True-Nuclear, catalog no. 421403) in the dark at room temperature. The antibodies used include granzyme B–FITC (catalog no. 515403, clone GB11), FOXP3–Alexa Fluor 532 (catalog no. 126407, clone MF-14), T cell factor 1 (TCF1)–Alexa Fluor 647 (catalog no. 6709, clone C63D9), the thymocyte selection-associated high mobility group box (TOX)–PE (catalog no. 12-6502-82, clone TXRX10), and Arg^1^-PE-Cy7 (catalog no. 105036, clone A1exF5). After staining, cells were analyzed using the Cytek Aurora flow cytometer. For mitochondrial staining, cells were washed with PBS and incubated with TMRM (100 nM; catalog no. T668) and MG (10 nM; catalog no. M7514) in PBS for 30 min in a 37°C incubator. Keima Green was excited with a wavelength of 488 nm and emitted at 615 nm, while Keima Red was excited with a wavelength of 561 nm and emitted at 615 nm. For live/dead staining, after mitochondrial staining and before cell surface marker staining, cells were incubated with LIVE/DEAD Fixable Blue (catalog no. L23105) in PBS on ice in the dark for 15 min.

### Human CBMCs

Human CBMCs were seeded into 96-well plates at 200,000 cells per well in RPMI medium supplemented with 10% FBS, 2 mM l-glutamine, penicillin-streptomycin, nonessential amino acids, 1 mM sodium pyruvate, 5 mM Hepes buffer, and β-mercaptoethanol. Cells were cultured in medium containing recombinant human IL-2 (100 IU/ml; PeproTech) and recombinant human IL-15 (20 ng/ml; PeproTech) and stimulated with antihuman CD3 (5 μg/ml, clone OKT3) and antihuman CD28 (1 μg/ml) antibodies. For the acute stimulation condition, anti-CD3/CD28 stimulation was applied only at the initiation of culture. For the chronic stimulation model, CBMCs were restimulated every 48 hours with anti-CD3/CD28 and fresh medium supplemented with IL-2 and IL-15 (*49*). Cells were maintained in culture for a total of 8 days. For USP30 inhibitor treatment, ST-539 (10 μM) was added on day 4 poststimulation and maintained throughout the remaining culture period. The cytotoxic potential was evaluated by intracellular granzyme B staining, and exhaustion markers were measured by surface staining for PD-1, Tim-3, and Lag3. The antibodies used include CD8-BV421(catalog no. 50165670, clone RPA-T8), PD-1–PE–Cy7 (catalog no. 25-9969-42, clone MIH4), TIM-3-BUV805 (catalog no. 569859, clone F38-2E2), LAG3-APC/Fire 750 (catalog no. 369214, clone 7H2C65), and granzyme B–AF700 (catalog no. 369214, clone GB11).

### CRISPR-Cas9–mediated gene deletion of murine CD8^+^ T cells

The CRISPR-Cas9–mediated KO procedure was adapted from a previously published method (*64*). Briefly, high-ranked guide sequences with the highest on-target and off-target scores were selected by CHOP-CHOP. Single guide RNA (sgRNA), Cas9 Nuclease V3, and Alt-R Cas9 Electroporation Enhancer were purchased from Integrated DNA Technologies (IDT) Inc. IDT sgRNA was resuspended in nuclease-free 1× Tris-EDTA (TE) buffer at 100 pmol/μl. Cas9/ribonucleoprotein (RNP) complex was assembled by combining Alt-R S.p. Cas9 Nuclease V3 with an NFATc1-targeting sgRNA (sequence: AAGCTGGTCATTATCACCGC) or USP30-targeting sgRNA (sequence: GGCGTCCAAGACCTCGTCCTCGG, TCAAACAAGTGGGTGACTCGAGG). Isolated CD8^+^ T cells were stimulated in 24-well plates precoated with anti-CD3 (3 μg/ml; 17A2) and anti-CD28 (1 μg/ml; 37.51) for 1 day. Two million activated CD8^+^ T cells were mixed with the Cas9/RNP complex in the P4 nucleofection buffer, along with Alt-R Cas9 Electroporation Enhancer. Electroporation was performed using the CM137 program on the Lonza 4D Nucleofector.

### Tumor xenograft and therapies

MC38 cells were cultured in DMEM with 10% FBS supplemented and 1% penicillin-streptomycin. Before injection, MC38 cells were diluted in PBS for 1 × 10^7^/ml. Tumor cell solution (100 μl) was injected subcutaneously into the mouse’s flank using insulin syringe. ST51000539 (ST-539) was purchased from TimTec. For in vitro experiment, ST-539 in dimethyl sulfoxide was added to the medium for a working concentration of 10 μg/ml. For in vivo experiment, ST-539 was homogenized in 20% sulfobutylether-β-cyclodextrin (Captisol) water for a final concentration of 5 mg/ml. Drug mixture (100 μl) was injected into the stomach of mice using oral gavage needle every other day.

### Confocal microscopy

Fluorescent samples were analyzed using a Zeiss LSM 780 confocal microscope (Carl Zeiss MicroImaging). Fluorescence of mt-Keima was captured in two channels with sequential excitations at 458 nm (Keima Green) and 561 nm (Keima Red), and emissions were collected within the range of 570 to 695 nm. Laser power was carefully optimized to ensure clear signal detection and maintained consistently across all experimental conditions.

### Seahorse assay

The mitochondrial function of CD8^+^ T cells was assessed using the Seahorse XF Cell Mito Stress Test Kit (Agilent) on an XF96 extracellular flux analyzer. Approximately 5 × 10^5^ cells were plated per well in an XF96 cell culture microplate. During the assay, mitochondrial stressors were sequentially injected at defined final concentrations, and the oxygen consumption rate was measured to evaluate key parameters of mitochondrial respiration.

### Western blotting

Samples were lysed in radioimmunoprecipitation assay buffer [50 mM tris-HCl, at pH 8.0, 150 mM NaCl, 1% (v/v) Nonidet P-40, 0.5% sodium deoxycholate, 0.1% SDS and protease inhibitor cocktail (Roche) on ice]. Primary antibodies were used at the following concentrations: p62/SQSTM1 (Abnova, H00008878-M01; 1:1000); OPTN (Proteintech, 10837-1-AP; 1:1000); LC3 (Cell Signaling Technology, 2775; 1:1000); glyceraldehyde-3-phosphate dehydrogenase (Cell Signaling Technology, 51332; 1:1000); and ACTIN (Cell Signaling Technology, 3700S; 1:1000). The membranes were incubated with anti-rabbit (LI-COR, 926-32211; 1:15,000) or anti-mouse (LI-COR, 926-68072; 1:15,000) immunoglobulin G secondary antibodies for 1 hour at room temperature. Images were captured using the Odyssey system (LI-COR).

### RNA extraction and cDNA synthesis

Total RNA was extracted from cells or tissues using TRIzol reagent (Direct-zol RNA MicroPrep, catalog no. R2062) according to the manufacturer’s instructions. The RNA concentration and purity were assessed using a NanoDrop spectrophotometer by measuring the absorbance at 260 nm and 280 nm. RNA with an A260/A280 ratio of ~2.0 was used for further experiments. Total RNA (1 μg) was treated with deoxyribonuclease I (Thermo Fisher Scientific) to remove genomic DNA contamination. First-strand cDNA was synthesized using a high-capacity cDNA reverse transcription kit (Applied Biosystems) with random hexamers as primers. The reaction was performed in a thermocycler at the following settings: 25°C for 2 min, 55°C for 10 min, and 95°C for 1 min. The synthesized cDNA was diluted 1:10 with nuclease-free water for qPCR.

### qPCR setup

qPCR reactions mix was performed in a total volume of 20 μl, consisting of 10 μl of SYBR Green Master Mix (Applied Biosystems or equivalent), 1 μl of forward primer (10 μM),1 μl of reverse primer (10 μM), 2 μl of cDNA template (diluted), and 6 μl of nuclease-free water. Forward primer of USP30: 5′-CTGTACAAAGATTGAAGCGAG-3′. Reverse primer of USP30: 5′-ACTGTTTAACAAAGGTGCTC′. qPCR was performed on a StepOnePlus Real-Time PCR System.

### Transcriptomics analysis

CD8^+^ T cells were isolated and cultured under acute or chronic TCR-mediated activation conditions, as described in Fig. 1A. Following stimulation, a total of 3 × 10^6^ cultured CD8^+^ T cells were flash-frozen in an ethanol/dry ice slush and shipped to the core facilities at Novogen, where library construction and sequencing were performed following standard protocols. The raw sequencing reads were trimmed using Trim Galore v0.6.10 and aligned to the reference genome (mm10) with HISAT2 v2.2.1 in combination with Samtools v1.2. Differentially expressed gene analysis was performed using DESeq2 v3.19. Gene set enrichment analysis (GSEA) was conducted with GSEA desktop software v4.3.3 in classic preranked mode. Data visualization included volcano plots generated with VolcaNoseR and heatmaps created using Morpheus (Broad Institute) and pheatmap v1.0.12.

### Reanalysis of USP30 expression in public datasets

To assess *USP30* expression in CD8^+^ T cells under different conditions, we reanalyzed publicly available transcriptomic datasets (*48*, *49*). Normalized read counts for *USP30* were extracted from the processed data and compared across experimental groups using a one-tailed Student’s *t* test to evaluate directional changes in expression. Statistical significance was defined as *P* < 0.05.

### Statistical analysis

Statistical analyses were performed in GraphPad Prism using unpaired or paired Student’s *t* tests for comparing two groups and one-way analysis of variance (ANOVA) with Tukey’s post hoc test for multiple group comparisons. The tumor growth curve was analyzed using two-way ANOVA with Tukey’s post hoc test. Specific statistical tests used for individual experiments are detailed in the figure legends.
