# Supplementary material for: USP30 inhibition augments mitophagy to prevent T cell exhaustion
Source: Sci Adv. 2025 Aug 15;11(33):eadv6902. doi: 10.1126/sciadv.adv6902 (PMC12356266; doi:10.1126/sciadv.adv6902)
Supplement: Supplementary file 1 — Figs. S1 to S7 [file sciadv.adv6902_sm.pdf]

Science Advances
AAAS

Supplementary Materials for

USP30 inhibition augments mitophagy to prevent T cell exhaustion

Ruohan Zhang *et al.*

Corresponding author: Nuo Sun, nuo.sun@osumc.edu; Gang Xin, gang.xin@osumc.edu

Sci. Adv. **11**, eadv6902 (2025)
DOI: 10.1126/sciadv.adv6902

This PDF file includes:

Figs. S1 to S7

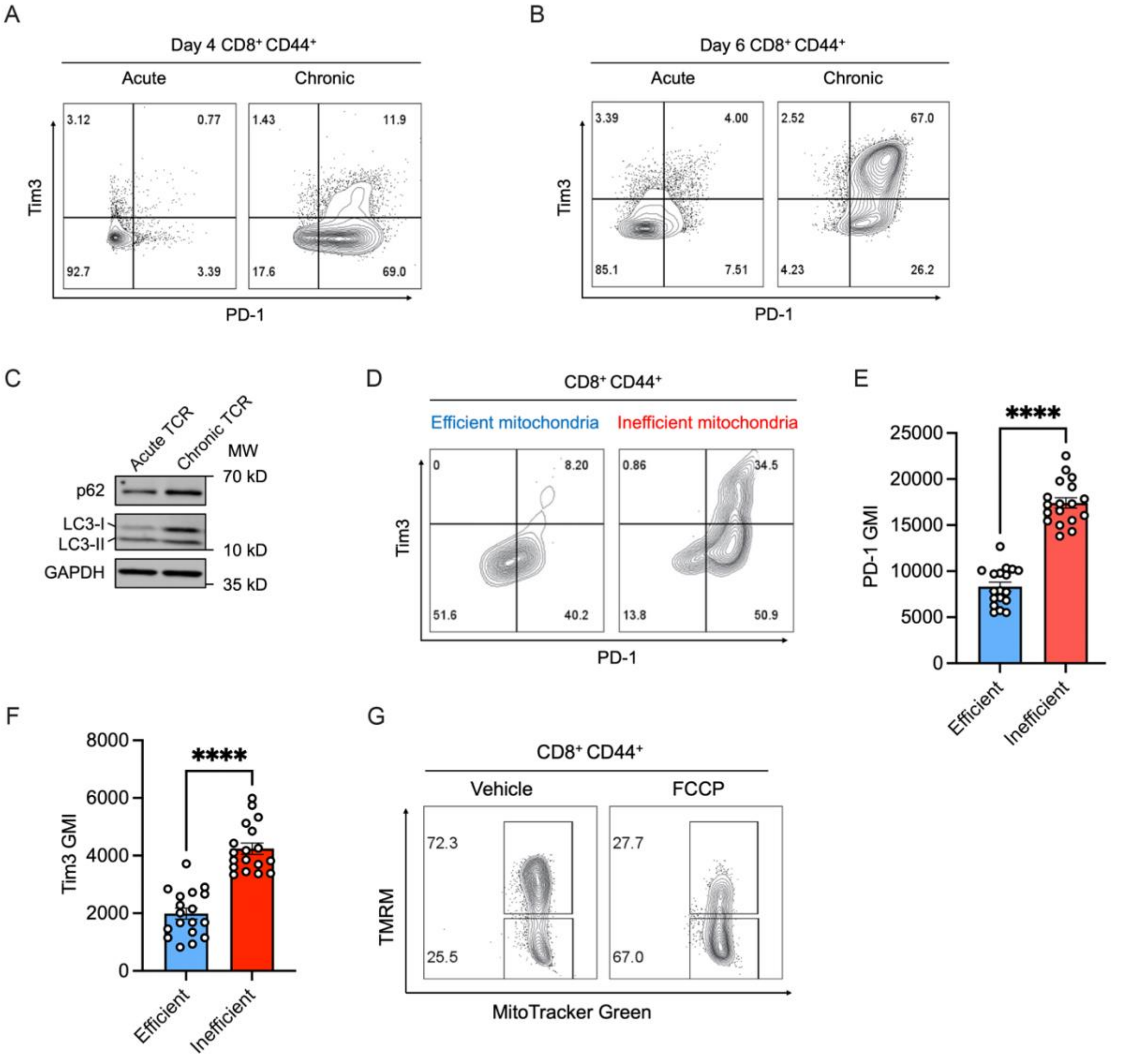

Fig. S1. Mitochondrial function is impaired in exhausted T cells.

(**A-B**) Representative flow cytometry plots showing Tim3 and PD-1 expression in CD8$^+$CD44$^+$ T cells following chronic and acute stimulation on **(A)** Day 4 and **(B)** Day 6. (**C**) Representative immunoblots of p62 and LC3-II/LC3-I protein expression in CD8$^+$ T cells following chronic and acute stimulation. Similar results were obtained in three biological replicates. (D) Representative flow cytometry plots showing Tim3 and PD-1 expression in CD8$^+$CD44$^+$ T cells with efficient or inefficient mitochondria (**E-F**) Quantification of PD-1 (**E**) and Tim3 (**F**) geometric mean intensity (GMI) in CD8$^+$CD44$^+$ T cells with efficient or inefficient mitochondria. Significance was calculated by unpaired t-test; ****$P < 0.0001$. Data are expressed as means ± SEM and the points correspond to the number of samples. (**G**) As a control condition, activated CD8$^+$ T cells were

treated with vehicle or Carbonyl cyanide-p-trifluoromethoxyphenylhydrazone (FCCP), a potent uncoupler that depolarizes mitochondrial membrane potential (MMP). Representative flow cytometry analysis of TMRM (MMP dependent) and MitoTracker Green (MMP independent) staining in $CD8^+CD44^+$ T cells.

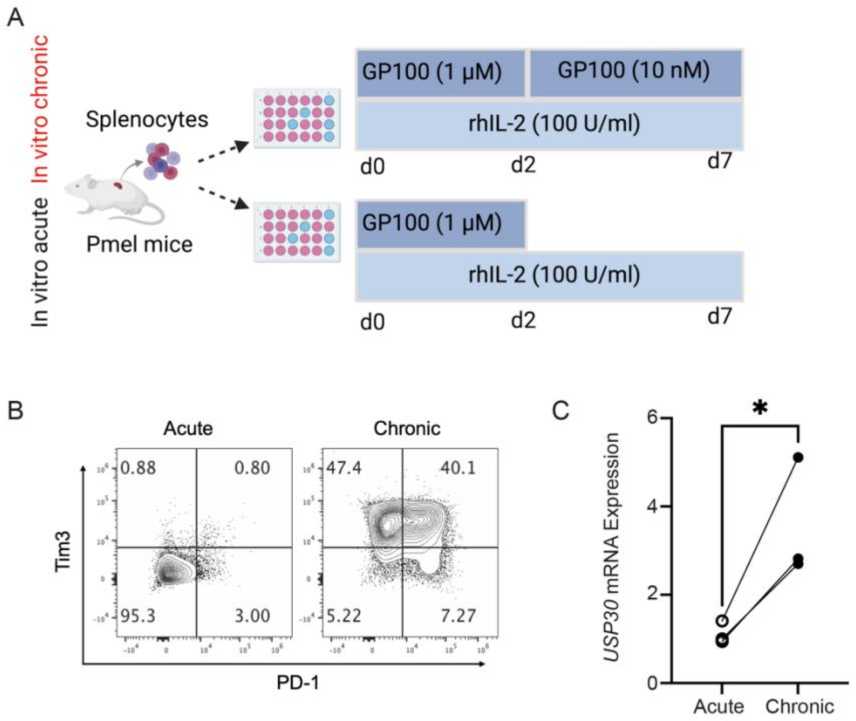

Fig. S2. Chronic antigen stimulation induces USP30 upregulation and exhaustion in antigen-specific CD8⁺ T cells.

(A) *In vitro* model of antigen-specific $CD8^+$ T cell exhaustion using Pmel-1 T cells. Splenocytes from Pmel-1 transgenic mice were stimulated with GP100 peptide (1 μM) and recombinant human IL-2 (100 U/mL) for 2 days. Cells were then cultured under either chronic stimulation conditions (GP100 10 nM + IL-2 100 U/mL) or acute stimulation conditions (IL-2 100 U/mL alone) for an additional 5 days. All cells were harvested on day 7. **(B)** Representative flow cytometry analysis of PD-1 and Tim3 expression in $CD8^+CD44^+$ T cells following acute or chronic antigen stimulation. **(C)** *USP30* mRNA expression in $CD8^+$ T cells from acute and chronic stimulation groups was quantified by qPCR (n = 3 biological replicates). *$P < 0.05$

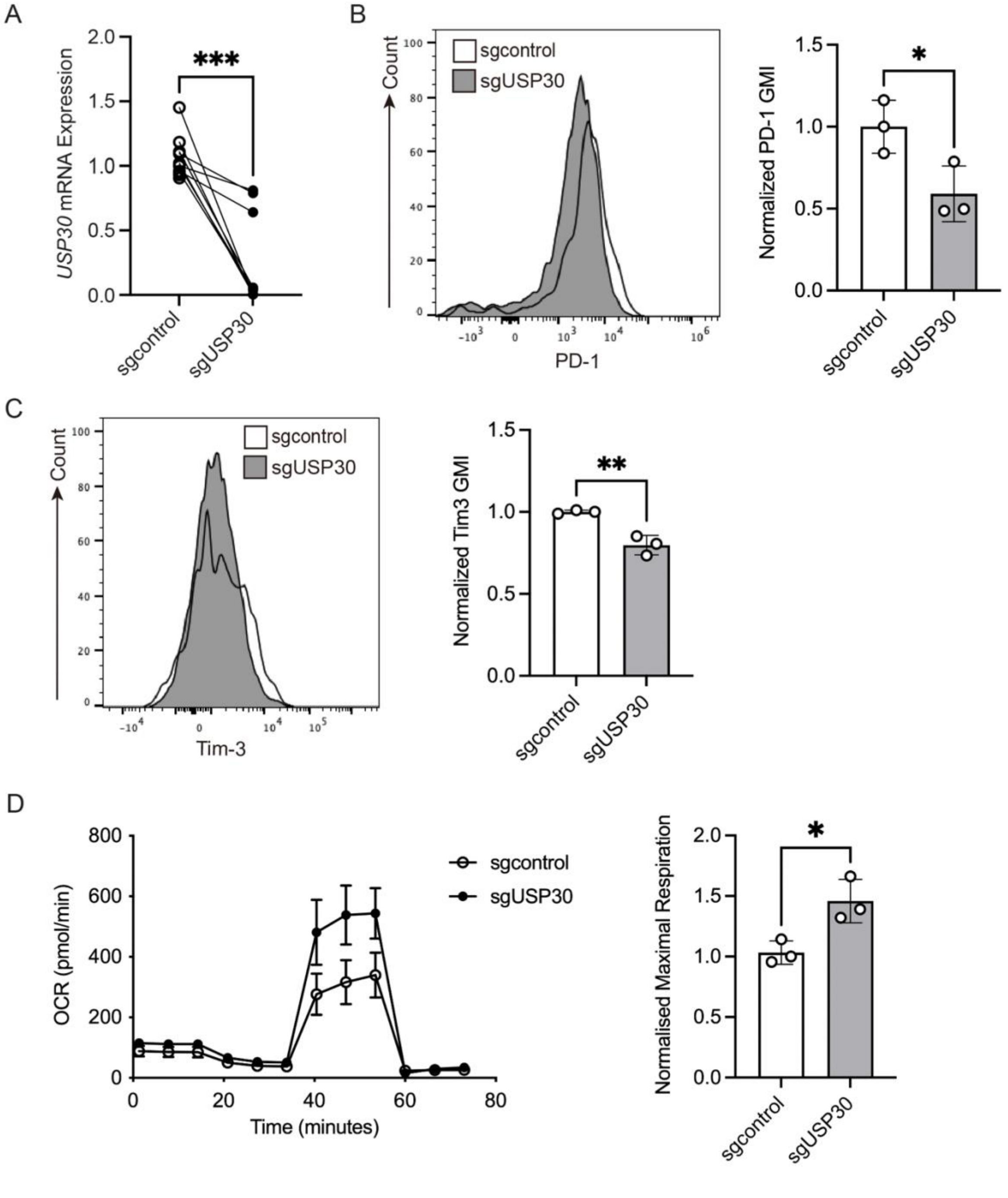

Fig. S3. USP30 deletion mitigates exhaustion in Pmel-1 T cells.

(A) CRISPR/Cas9-mediated knockout of USP30 in Pmel-1 T cells. Cells were electroporated with either non-targeting control Cas9 RNP (sgcontrol) or a guide RNA targeting *USP30* (sgUSP30). *USP30* mRNA expression was measured by RT-qPCR 72 hours after *in vitro* activation (n = 6). ***$P < 0.001$. (B, C) Representative flow cytometry plots and quantification of PD-1 **(B)** and Tim3

(C) expression in $CD8^+CD44^+$ T cells under chronic antigen stimulation. Data are presented as mean ± SD; n = 3. *$P < 0.05$, **$P < 0.01$. **(D)** Seahorse Mito Stress Test showing oxygen consumption rate (OCR) in sgcontrol and sgUSP30 $CD8^+$ T cells. The line graph shows OCR response to sequential injection of mitochondrial inhibitors; the bar graph quantifies maximal respiratory capacity. Data represent cumulative results from three independent experiments (n = 3). *$P < 0.05$.

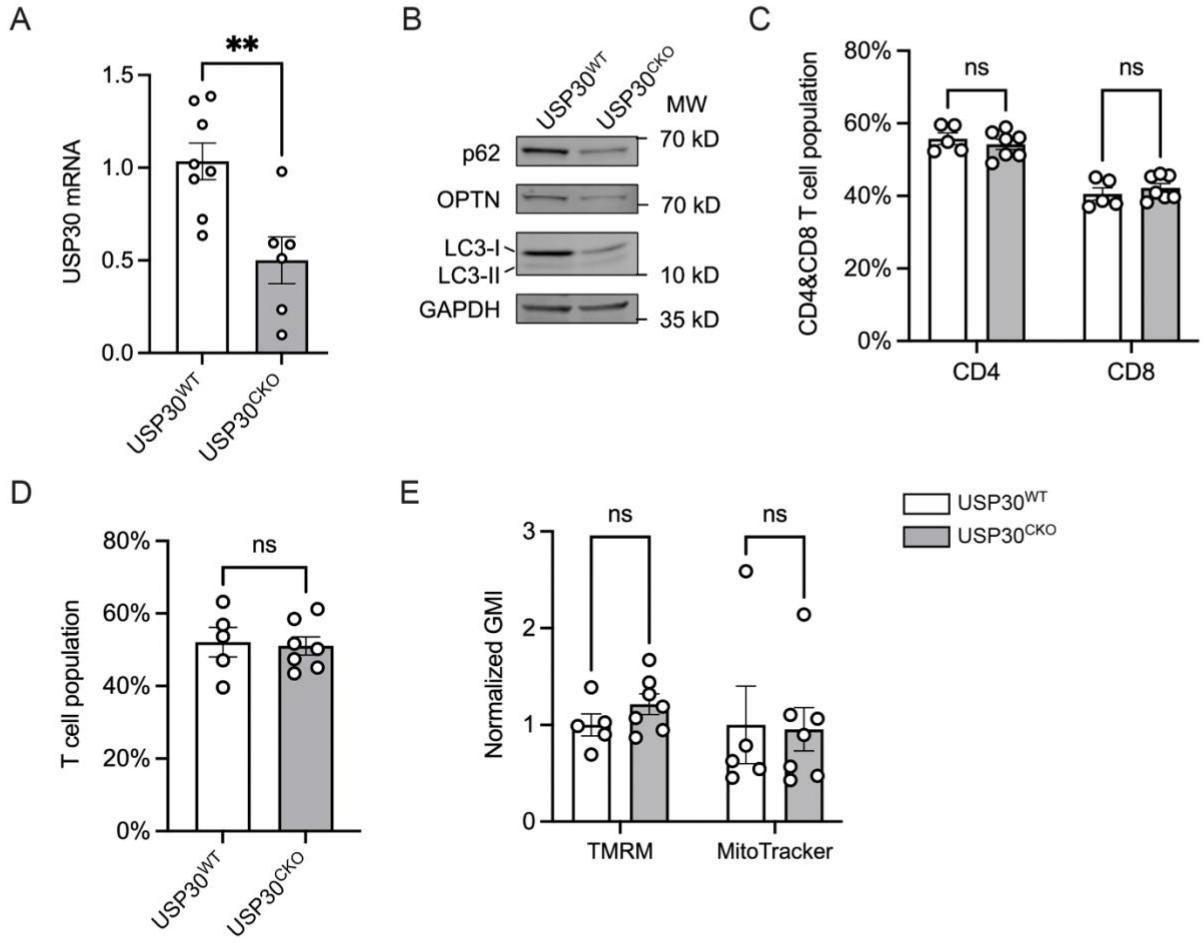

Fig. S4. USP30 deletion does not significantly affect T cells outside the tumor microenvironment.

(A) Quantification of USP30 expression in USP30^{WT} and USP30^{CKO} CD8^{+} T cells. Significance was determined using an unpaired t-test; $^{**}P < 0.01$. **(B)** Representative immunoblots of p62, OPTN, and LC-II/LC-I protein expression in CD8^{+} T cells from USP30^{WT} and USP30^{CKO} mice under chronic stimulation. Similar results were obtained in three biological replicates. **(C)** The proportions of splenic CD4^{+} and CD8^{+} T cells from naïve mice. **(D-E)** MC38 murine colon adenocarcinoma cells were inoculated into USP30^{WT} and USP30^{CKO} mice. **(D)** total T cell populations, and **(E)** normalized geometric mean intensity (GMI) of TMRM and MitoTracker staining in splenic CD8^{+} T cells were analyzed by flow cytometry. Significance was assessed by unpaired t-test, ns, not significant.

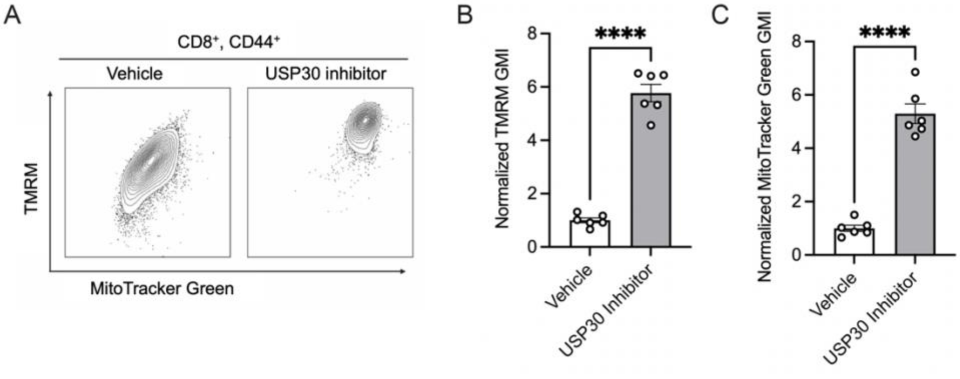

Fig. S5. USP30 inhibition improves mitochondrial activity in $CD8^+$ T cells.

(A) Representative flow cytometry plots showing TMRM and MitoTracker Green staining in $CD8^+CD44^+$ T cells treated with vehicle or USP30 inhibitor. **(B, C)** Quantification of TMRM **(B)** and MitoTracker Green **(C)** geometric mean intensity (GMI) in $CD8^+CD44^+$ T cells treated with vehicle or USP30 inhibitor. Significance was calculated by unpaired t-test; ****$P < 0.0001$

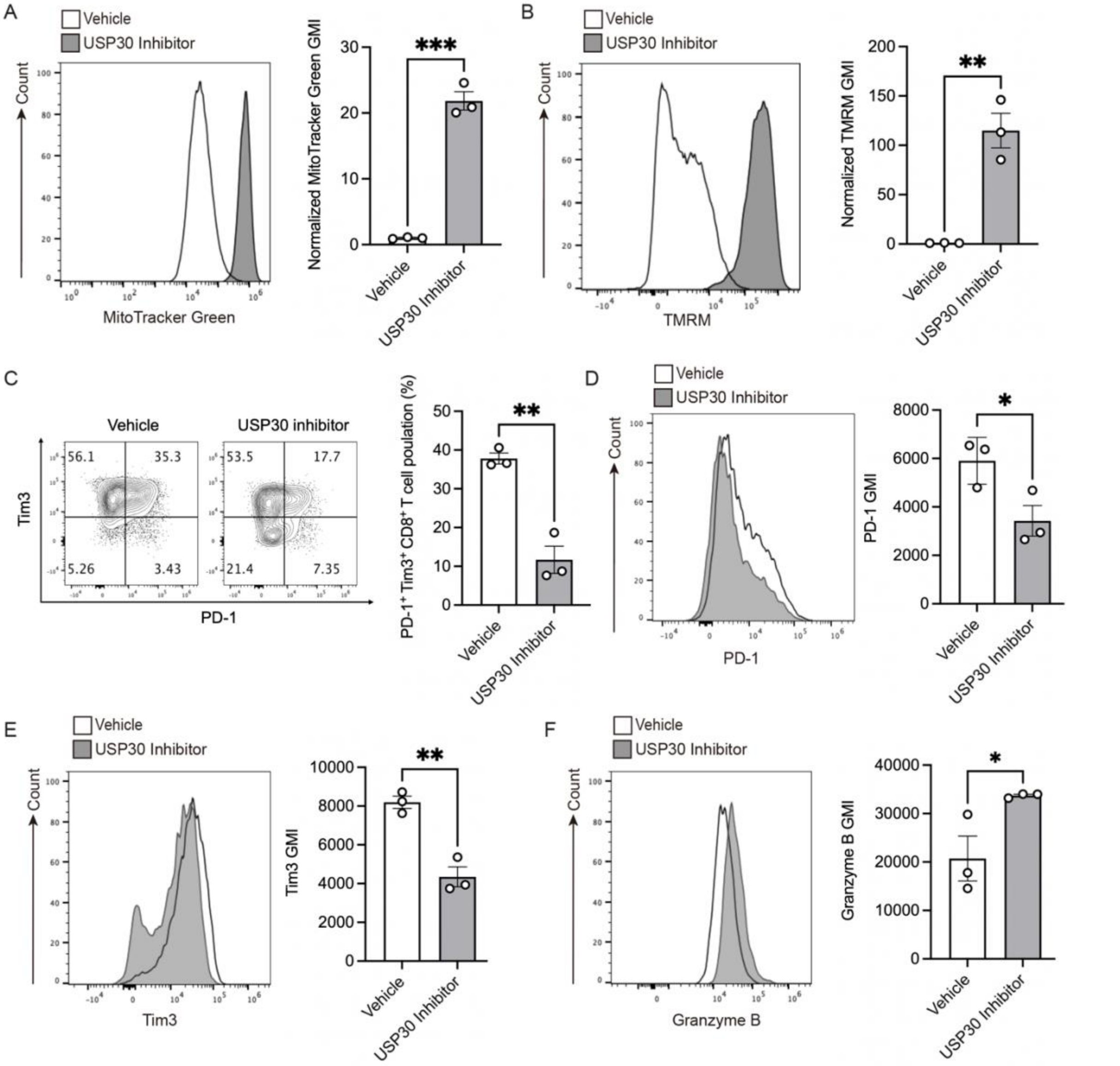

Fig. S6. USP30 inhibition enhances mitochondrial fitness and effector function in chronically stimulated Pmel-1 T cells.

(A–F) Pmel-1 T cells were chronically stimulated with GP100 peptide in the presence of vehicle or USP30 inhibitor (ST-539). On day 7, cells were harvested and analyzed by flow cytometry. (A, **(B)** Representative flow cytometry plots and quantification of MitoTracker Green **(A)** and TMRM **(B)** in $CD8^{+}CD44^{+}$ T cells. **(C)** Representative flow cytometry plots and quantification of $PD\text{-}1^{+}$ $Tim3^{+}$ subsets. **(D, E)** Representative plots and quantification of PD-1 **(D)** and Tim3 **(E)** expression in $CD8^{+}CD44^{+}$ T cells. **(F)** Normalized flow cytometry analysis of Granzyme B

expression. Significance was determined by unpaired t-test (n = 3); Data represent means ± SEM and the points correspond to the number of samples. $^*P < 0.05$, $^{**}P < 0.01$.

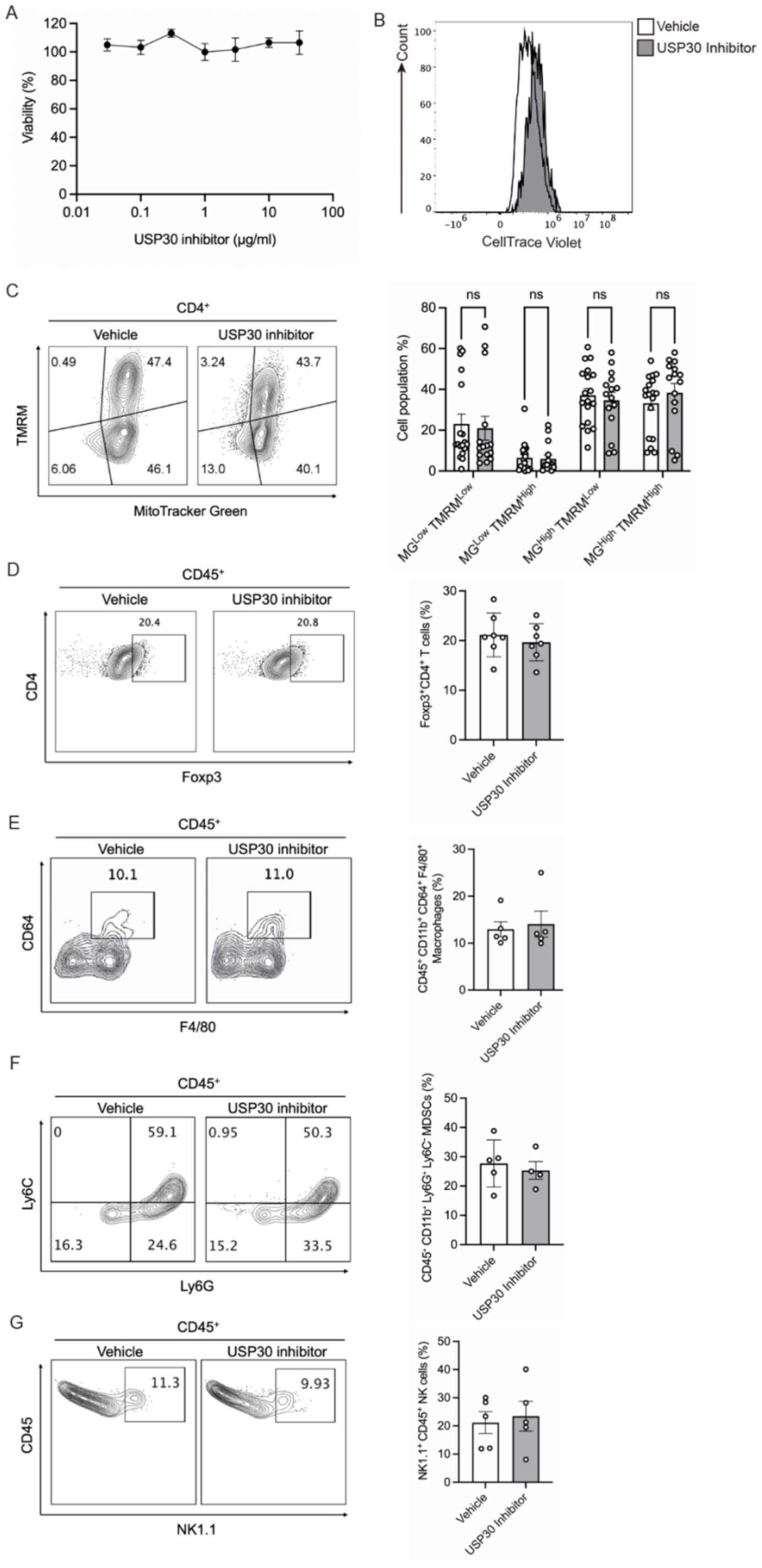
A
Viability (%)
USP30 inhibitor (μg/ml)
B
Vehicle
USP30 Inhibitor
Count
CellTrace Violet
C
CD4⁺
Vehicle
USP30 inhibitor
0.49
47.4
6.06
46.1
3.24
43.7
13.0
40.1
TMRM
MitoTracker Green
ns
Cell population %)
MG^Low TMRM^Low
MG^Low TMRM^High
MG^High TMRM^Low
MG^High TMRM^High
D
CD45⁺
Vehicle
USP30 inhibitor
20.4
20.8
CD4
Foxp3
Foxp3⁺CD4⁺ T cells (%)
Vehicle
USP30 Inhibitor
E
CD45⁺
Vehicle
USP30 inhibitor
10.1
11.0
CD64
F4/80
CD45⁺ CD11b⁺ CD64⁺ F4/80⁺ Macrophages (%)
Vehicle
USP30 Inhibitor
F
CD45⁺
Vehicle
USP30 inhibitor
0
59.1
16.3
24.6
0.95
50.3
15.2
33.5
Ly6C
Ly6G
CD45⁺ CD11b⁺ Ly6G⁺ Ly6C⁻ MDSCs (%)
Vehicle
USP30 Inhibitor
G
CD45⁺
Vehicle
USP30 inhibitor
11.3
9.93
CD45
NK1.1
NK1.1⁺ CD45⁺ NK cells (%)
Vehicle
USP30 Inhibitor

Fig. S7. USP30 inhibition does not impact tumor cell proliferation *in vitro* or non-T cell compartments in the tumor microenvironment *in vivo*.

(A) Dose-response curves of USP30 inhibitor (ST-539) on murine MC38 colorectal cancer cells (n = 3). **(B)** T cell expansion following TCR activation in the presence or absence of ST-539. **(C)** MC38 tumor-bearing mice were treated with vehicle or ST-539 every other day, and tumor-associated $CD4^+$ T cells were analyzed by flow cytometry. Representative flow cytometry plots and quantification show mitochondrial efficiency in $CD4^+$ T cells, assessed by TMRM and MitoTracker Green (MG) staining, with the proportion of each mitochondrial subpopulation indicated. **(D)** Representative flow cytometry plots and quantification of Foxp3 expression in $CD4^+$ T cells. **(E-G)** MC38 tumor-bearing mice were treated with vehicle or ST-539 for 14 days. Tumor-infiltrating immune cells were isolated and analyzed by flow cytometry. (E) Tumor-associated macrophages (TAMs): $CD45^+CD11b^+CD64^+F4/80^+$. **(F)** Myeloid-derived suppressor cells (MDSCs): $CD45^+CD11b^+Ly6G^+Ly6C^-$. **(G)** Natural killer (NK) cells: $CD45^+NK1.1^+$. Percentages of each population among $CD45^+$ cells are shown. Data represent means ± SEM and the points correspond to the number of samples. Statistical significance was assessed by unpaired t-
